# High-throughput toxicity study of lubricant emulsions and their common ingredients using zebrafish

**DOI:** 10.1371/journal.pone.0207946

**Published:** 2018-11-21

**Authors:** Jae-Hoon Han, Sang-Kyu Jung

**Affiliations:** Department of Bio. & Chemical Engineering, Hongik University, Sejong, S. Korea; Institute of Materials Science, GERMANY

## Abstract

Though lubricant emulsions have been widely used in many industrial processes, various human health hazards have been reported. Conducting a systematic toxicity study on emulsions is difficult since emulsions contain multiple chemical compounds, and hydrophobic compounds form complex emulsion particles via surfactants. For a quantitative toxicity study, we developed a high-throughput imaging system using zebrafish and conducted a large scale in vivo toxicity assay of lubricant emulsion and their common ingredients. By computing the locomotion activity of zebrafish from captured time-lapse images, we could quantify the degree of relative toxicity of 29 chemicals. The changes in the locomotion activity over time were observed to vary significantly depending on emulsions, indicating that the degree of toxicity of the commercial products was very diverse. We found that primary ethanolamines were more toxic than secondary or tertiary ethanolamines, and several factors, such as alkyl chain length, EO mole, test concentration, and emulsion particle size, affected toxicity.

## Introduction

A lubricant emulsion is generally defined as a mixture of two or more liquid substances that are normally immiscible for improved lubricity and cooling properties. In various industrial processes, lubricant emulsions (or simply emulsions) have been used frequently for efficient metal cutting, sharpening, and material processing. For example, metalworking emulsions are the most important type of emulsions; it is estimated that the global metalworking fluid market was USD 9.23 billion in 2014, and in the USA alone, about 375 kilotons of metalworking fluid were used in 2017 [1]. However, various human health hazards have been reported. Contact dermatitis, asthma, lung irritation, and hypersensitivity pneumonitis are associated with emulsion exposures [2, 3]. There is significant evidence that emulsions significantly increase the risk of certain cancers, such as lung, liver, pancreatic, skin, laryngeal, and leukemia, and testicular [2, 4]. Moreover, emulsions can easily contaminate soil and water accidentally, damaging animals that live in these areas. Thus, the environmental impacts caused by emulsions must be properly assessed.

However, there have been few systematic and extensive toxicity studies on emulsions since emulsions are composed of a wide variety of compounds and can have very different physicochemical and biological properties. Specifically, emulsions are complex water-miscible fluids that include components such as base oils, amines, fatty acids, surfactants, anti-rust agents, long chain alcohols, extreme pressure agents, and biocides [5]. Commercial emulsion products can contain as many as 10–20 different compounds that may exhibit their own inherent toxicity. Moreover, in emulsions, hydrophobic materials form small micelles or colloids dispersed in water by surfactants, and the size and stability of emulsions can be greatly changed by a tiny amount of surfactant. These changes in emulsion properties may also further modify the overall toxicity of emulsions conferred by raw materials. Therefore, toxicity studies on emulsions require the development and usage of methods that allow for fast and affordable high-throughput experiments on a large number of combinations of materials and properties. However, typical toxicity assays using whole animals are usually expensive and require a long period of experimentation.

Recently, image-based toxicity assays using small animal models have been developed and used for large-scale toxicity studies [6–10]. Compared to traditional cell-based assays, these technologies allow intact whole animals to be tested and can detect organism-level toxicity [11]. There are many reports demonstrating that quantifying animal phenotype or behavior through image analysis is a good way to measure the degree of toxicity. We previously developed a high-throughput imaging platform called QuantWorm and successfully quantified the toxicities of 20 different nanomaterials using 1 mm long *Caenorhabditis elegans* [11, 12]. Zebrafish will be a good animal model for toxicity studies as reported in the literature [6–8, 10, 13–17]. As an advanced vertebrate, zebrafish has a variety of organs similar to humans, including a brain capable of complex behavior, whereas *C*. *elegans* have simple nervous systems. In image analysis, compared to *C*. *elegans*, zebrafish larva is very large in size, so it can be easily imaged with economic camera devices without requiring a separate microscope.

In this article, we developed an automated imaging system to quantify the locomotion activity of zebrafish and measured the individual acute toxicities of 20 different amines and surfactants commonly used in the commercial emulsion as well as 9 emulsion products. The toxicity analysis of individual raw materials will provide an important basis for selecting raw materials when developing commercial emulsions with reduced toxicity. For this study, a new simple locomotion activity was used, and different ages of zebrafish (adult vs. larvae) and concentrations of chemicals were also examined. To our knowledge, this is the first large-scale toxicity study of emulsions and their common ingredients using intact whole animals.

## Materials and methods

### Chemicals and plates

Most test chemicals, including emulsions, amines, and surfactants, were kindly donated by Korea Houghton Co., Ltd. or purchased from commercial distributors in Table 1. Six commercial emulsions (Oil-A, -B, -C, -D, -E, and -F) were originally 3% (Brix) diluent, and their names were written in alphabetical order for anonymity in Table 2. Three TMP emulsions (TMP-72.5, -77.5 and -82.5) were manufactured directly. All chemicals were kept at 25 °C in a dark room and diluted in filtered water and homogenized for 30 seconds prior to toxicity tests. To anesthetize zebrafish larva, 0.6 mM tricaine (ethyl 3-aminobenzoate methanesulfonate, Sigma, Catalog No. A5040) was used.

**Table 1:** List of chemicals.

Category	Abbreviation	Chemicals
**Amine**	**MEA**	Monoethanolamine (2-aminoethan-1-ol)
	**DEA**	Diethanolamine (2,2'-iminodiethanol)
	**TEA**	Triethanolamine (2,2',2''-nitrilotri(ethan-1-ol)
	**MIPA**	Monoisopropanolamine (1-aminopropan-2-ol)
	**DGA**	Diglycolamine (2-(2-aminoethoxy)ethanol)
	**AMP95**	2-Amino-2-methyl-1-propanol (95%)
	**AMP75**	2-Amino-2-methyl-1-propanol (75%)
	**MDEA**	Methyldiethanolamine
	**BDEA**	Butyldiethanolamine
	**TPT**	Trimethylolpropane polyoxypropylene triamine
	**CHA**	Cyclohexyldiethanolamine
	**AB**	2-Amino-1-butanol (67%), 2-Amino-2-ethyl-1,3-propanediol (14%)
	**AEP**	2-Amino-2-ethyl-1,3-propanediol (78%), 2-Amino-1-butanol (5%)
**Surfactant**	**Tween81**	Tween 81
	**EO/PO**	Propylene oxide and ethylene oxide block copolymer
	**CFE**	Polyoxyethylene coconut fatty ester (EO 5.5 mole)
	**KREL10**	Polyoxyethylene castor ether (EO 10 mole)
	**KREL12**	Polyoxyethylene castor ether (EO 12 mole)
	**KREL15**	Polyoxyethylene castor ether (EO 15 mole)
	**KREL20**	Polyoxyethylene castor ether (EO 20 mole)

**Table 2:** List of emulsions.

	Emulsions	Specific gravity (15/4°C)	Surface tension^a^ (dyne/cm)	TAN^b^ (mg KOH/g)	Median particle size (D50)
**Oil-A**	Commercial anionic emulsion (Nica Chemical, Japan)	0.9728	28.7	0.2	313 nm
**Oil-B**	Commercial cationic emulsion (Korea Houghton, Korea)	0.9906	27.8	2.3	1.04 μm
**Oil-C**	Commercial anionic emulsion (Schill+Seilacher GmbH, Germany)	1.0025	31.4	0.9	5.67 nm
**Oil-D**	Commercial nonionic emulsion (Korea Houghton, Korea)	1.0603	30.5	5.2	1.12 μm
**Oil-E**	Commercial nonionic and anionic emulsion (Sanyo Chemical Industries, Japan)	0.9970	33.5	5.8	14.5 nm
**Oil-F**	Commercial nonionic and anionic emulsion (Sanyo Chemical Industries, Japan)	0.9843	29.3	10.9	239 nm
**TMP-72.5**	Trimethylolpropane trioleate (72.5%) + EO/PO surfactant (27.5%)	0.9381	38.7	2.58	168 nm
**TMP-77.5**	Trimethylolpropane trioleate (77.5%) + EO/PO surfactant (22.5%)	0.9422	38.6	2.76	158 nm
**TMP-82.5**	Trimethylolpropane trioleate (82.5%) + EO/PO surfactant (17.5%)	0.9462	37.9	2.94	231 nm

^a^Surface tension was measured at 3% (Brix) in water.

^b^Total Acid Number

To test zebrafish larvae, we used transparent 15-well spot plates with concave depressions that measured 20 mm × 20 mm × 10 mm (length × width × height) for each well (Canada, FLINN Scientific, Item No. AP6404). To test adult zebrafish, we used 8-well grating squared chambers where each well measured 60 mm × 80 mm × 30 mm (S. Korea, Changsin Living, Item No. CSNGNM015406).

### Chemical analysis

The pH of the chemicals was measured using the equipment (Core Parmer, Model 3510). Specific gravity and total acid number (TAN) values were measured as described by the ASTM D 1298 and ASTM D 664/974, respectively. Surface tension was measured by the ring method using a Du Noüy ring connected to a KSV Sigma 701 (Finland). The particle size distribution was analyzed using a particle size analyzer (K-ONE Nano, Scatteroscope 55).

### Animals

Wild-type zebrafish larvae were obtained from the Korean Zebrafish Organogenesis Mutant Bank (ZOMB). Zebrafish were maintained at 28 °C in day and night cycles (14 hr: 10 hr) using LED lights. Zebrafish larvae at 14–19 days post-fertilization (dpf) and adult zebrafish that were about six months old were used for toxicity experiments. Photos of zebrafish larvae were taken using an Olympus SZX7 stereo-microscope and digital camera (DigiRetina16) after anesthetizing them with 0.6 mM tricaine. For adult zebrafish, images were taken after quick freezing.

### Chemical exposure

Animal toxicity experiments were carried out in a collaborative lab at Inha University, and all animal experiments using zebrafish were approved by the Inha University Animal Care and Use Committee (IACUC) under protocol (INHA 180821–588).

Chemical exposure was carried out on the 15-well spot plates for zebrafish larvae or the 8-well grating chamber for adult zebrafish. Toxicity tests were initiated by filling each empty well with fresh water (70 mL for adult and 0.5 mL for larva). After transferring zebrafish to each well, animals were allowed to adapt for 1 hour. Diluted test chemical solutions were then dropped onto each well and imaging began. The final working volumes per well were 75 mL and 1 mL for adult zebrafish and zebrafish larvae, respectively. Unless otherwise specified, each well in the multi-well plates contained one animal and the test solution included 0.5% (v/v) of the prepared sample. Test solutions of amines, surfactants, and emulsions had a pH of about 10, 5, and 5.5, respectively.

### Statistical analysis

All experiments were performed independently at least three times. Mean activity was shown as the mean and standard deviation. The locomotion activity of the animals tested under the same conditions was averaged every 5 minutes after being combined and then used for statistical analysis. A one-way ANOVA test followed by Tukey’s multiple comparison test was conducted using SigmaPlot software to assess the significance of toxicity differences between conditions. At least five zebrafish were tested under each condition, and statistical analysis was performed on all zebrafish except for the one with the largest variation in activity.

## Results and discussion

### Characterization of emulsions

Important properties of emulsions such as pH, specific gravity, viscosity, total acid number (TAN), and particle size distribution were analyzed in Table 2. Every emulsion had one major peak in volume-based particle size distribution as shown in S1 Fig. Oil-B and -D had a large particle size of about 1 μm, while Oil-A, -C, -E, -F had a size of 5.67 nm to 313 nm. TMP-82.5 had a larger emulsion particle size (D50 = 231 nm) than TMP-72.5 (D50 = 168 nm) and TMP-77.5 (D50 = 158 nm). The effect of emulsion particle size on toxicity will be discussed later.

### Development of imaging hardware and analysis algorithm

**Fig 1:**
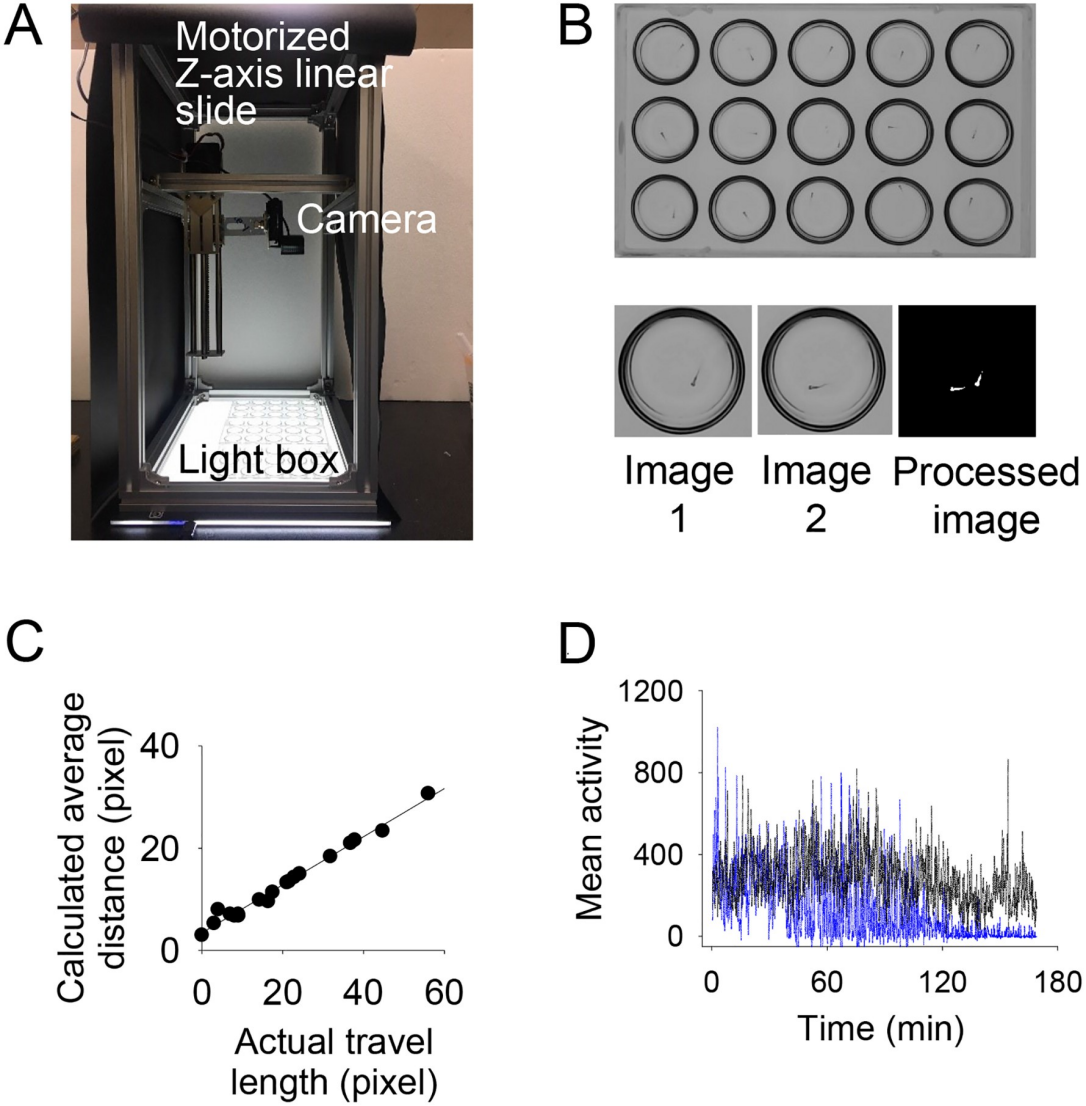
An imaging device was developed, and analysis software was created to evaluate chemical toxicity by quantifying the movements of zebrafish. (A) The imaging device consists of a computer-controlled z-axis linear slide, camera, spot plate, and flat light box. (B) Image analysis software creates binarized subtracted images from two time-lapse images and computes locomotion activity. (C) Correlation between the calculated average distances of white pixels in binarized images and actual travel lengths of animals (R^2^ = 0.9854). (D) Difference in locomotion activity between the control (black) and TPT-treated animals (blue) over time.

To measure the effects of emulsions and their common ingredients on zebrafish, we developed a bright field imaging system that was composed of a digital camera (Logitech C920), a 150 mm-long motorized Z-axis linear slider, a multi-well spot plate, and a light plate in Fig 1A. The position of the motorized linear slider was controlled by an Arduino Nano board and a custom computer program that adjusted the distance between the spot plate and the camera. Images were automatically taken every 1 second for 3 hours using PhenoCapture (www.phenocapture.net). Since the multi-well spot plate is transparent and has rounded concave depressions without vertically extended walls, the whole area of the well is imaged properly without being blocked by the wall of the well even if the camera’s field of view is slightly offset in Fig 1B.

The degree of toxicity of individual chemicals was quantified by calculating locomotion activities from the captured images. Locomotion activity was originally defined as the number of white pixels in the binarized differential image of two time-lapse images [18]. However, although it is advantageous that locomotion activity is capable of grasping the movements of an animal through simple calculations without using object tracking techniques, it does not reflect how far the animal has moved from its original position should the animal leave its initial position.

To overcome this shortcoming, we defined a new locomotion activity and developed a simple image processing algorithm to quantify the degree of motion of individual zebrafish. The new locomotion activity was defined as the product of the original locomotion activity and the average distance between all the white pixels in the binarized differential image.

Automated image processing was performed using home-made image analysis software written in VB.NET in S2 Fig. The first step in image processing is to obtain a differential image from two successive images through pixel-by-pixel subtraction [12, 18]. In the case of the adult zebrafish, we used a box averaging filter (also called blur filter or neighborhood averaging filter) to reduce noise before subtraction. In the case of the zebrafish larva, we did not normally apply this filter. The differential image was then binarized by an adaptive local thresholding algorithm with a threshold value of 83% [19]. Through this processing, the animal’s movements were displayed as white pixels. Since single white pixels affected the locomotion activity, they were removed.

To compute the improved locomotion activity, the software first counts the number of white pixels in the binarized image. To compute the average distance between white pixels, straight-line distances were calculated by selecting two white pixels from all white pixels, and then the distances obtained for all combinations of two white pixels were averaged. The improved locomotion activity was finally calculated by multiplying the number of white pixels and the average distance between white pixels. Various sample images and analysis results were shown in S3 Fig.

After testing the collected images, it was found that there were good linear correlations between the actual travel lengths of animals and the calculated average distances of white pixels in the binarized differential images (R^2^ = 0.9854) in Fig 1C and between actual travel lengths and improved locomotion activities (R^2^ = 0.9863), whereas the original locomotion activity became saturated after a certain measured travel length and did not change after that point in S4 Fig. These results represent that the improved locomotion activity not only captures the difference in animal posture or motion, but also quantifies the distance that the animal had moved. This method is useful because it is applicable to blurry images and does not require complex object tracking techniques. Unless otherwise noted, in this article, the improved locomotion activity is simply referred to as the activity or locomotion activity.

An exemplary analysis result of the images obtained from the toxicity test is shown in Fig 1D. Though both a control larval zebrafish and test larvae treated with TPT made irregular movements, the locomotion activity level of the control fish was much lower than that of the test animal. However, the test fish showed little movement after 2 hours. In fact, when locomotion activity stopped, it was confirmed that the test fish had died.

### Zebrafish were very sensitive to toxicity of emulsions

**Fig 2:**
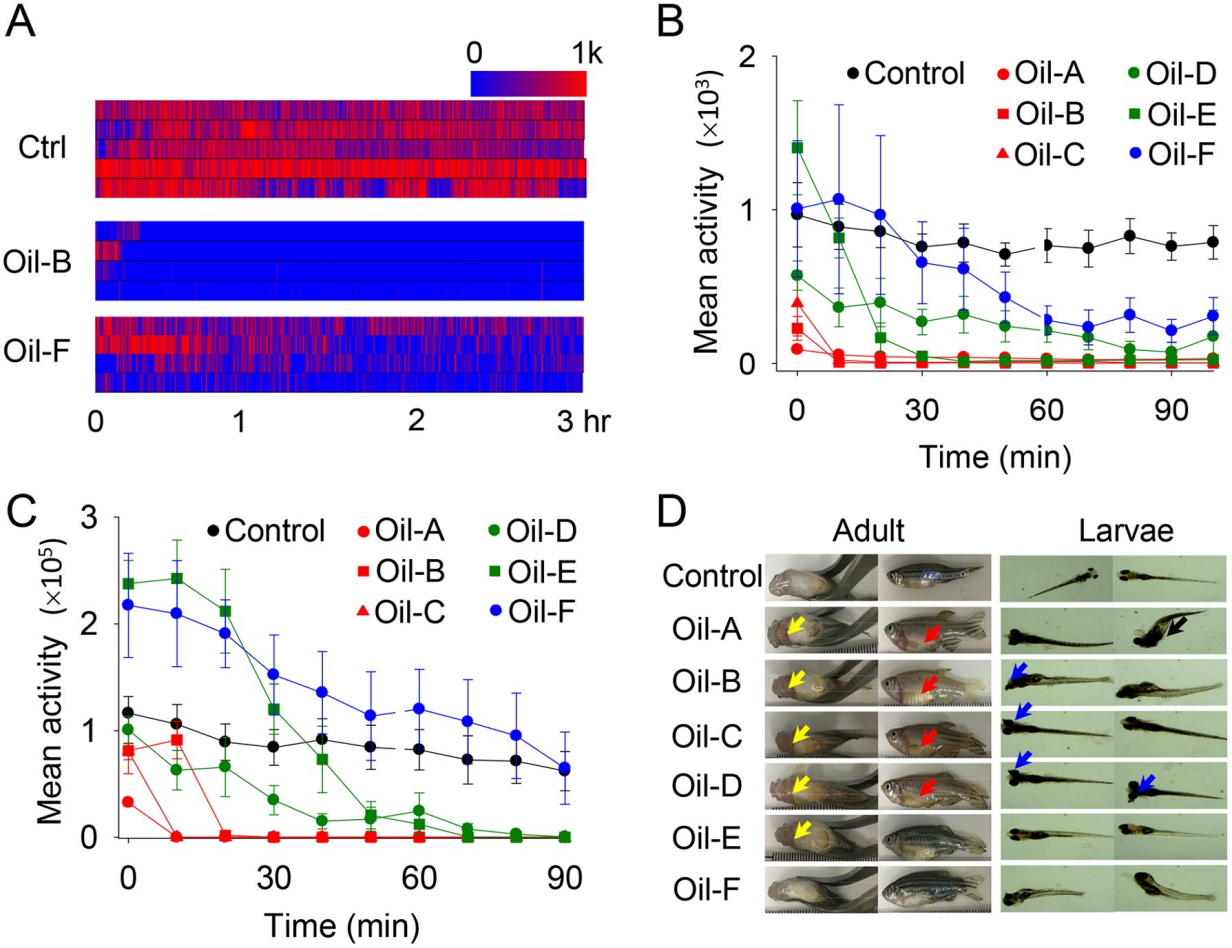
Toxicity tests of commercial emulsions showed their various effects on locomotion activity. (A) Heat map of the locomotion activity of individual larval zebrafish over time. Each row represents a different zebrafish. (B) Effect of emulsions on locomotion activity in larvae (14 dpf). Control: black circle; Oil-A: red circle; Oil-B: red square; Oil-C: red triangle; Oil-D: green circle; Oil-E: green square; Oil-F: blue circle. The locomotion activity of all animals tested under the same condition was recorded as a mean value for 10 minutes. Sample size: *n* = 5 animals (one animal per well). (C) Effect of emulsions on locomotion activity in adult zebrafish. Control: black circle; Oil-A: red circle; Oil-B: red square; Oil-C: red triangle; Oil-D: green circle; Oil-E: green square; Oil-F: blue circle. Sample size: *n* = 5 animals. (D) Phenotype observation of animals after three hours of exposure. Yellow arrow: internal hemorrhaging around the heart and gills; Red arrow: skin damage; Blue arrow: eyeball damage; Black arrow: body distortion. Oil-E and Oil-F did not show any noticeable differences.

Toxicity experiments were conducted on six commercial emulsion products using multiple adult or larval zebrafish in Fig 2.

A heat map of locomotion activity in individual worms over time was shown in Fig 2A. We observed a similar degree of locomotion activity among animals treated with the same compound as well as distinguishable locomotion activity among other compounds. This result indicates that quantifying the degree of toxicity of emulsions by locomotion activity was highly reliable.

The relative toxicity of the tested emulsions was then compared with the average locomotion activity over 10 minutes. Each compound exhibited a very different profile of locomotion activity over time, with a higher activity for Oil-A, -B and -C, with -F having the least activity in Fig 2B and 2C. This trend was similar for both larval and adult zebrafish. In addition, most of the samples showed no movement between 60–90 minutes because they had died. Similar results were observed for both adult and larval fish.

The degree of relative toxicity measured from their movements was generally consistent with the observed phenotype results. Although zebrafish treated with Oil-E and -F initially showed hypersensitivity, no serious harm to the body was observed in Fig 2D. On the contrary, zebrafish treated with Oil-A, -B, -C, and -D suffered fatal damages, such as infiltrated skin layers and hyperemia around heart and gills, possibly caused by defects in cardiovascular systems and skin tissues. In zebrafish larva, more severe symptoms, such as a distorted body, torn skin tissue, and impaired eyeballs were seen for the same concentrations. Perhaps the scales of larval zebrafish are not fully developed, and their bodies are vulnerable, making them more easily affected by the chemicals.

The result of the changes in the locomotion activity over time was observed to vary widely depending on emulsions, indicating that the relative toxicity of commercial emulsion products is diverse and the effects of toxicity on human health and the environment may be very wide.

### Various degrees of toxicity were detected from major constituent amines and surfactants

**Fig 3:**
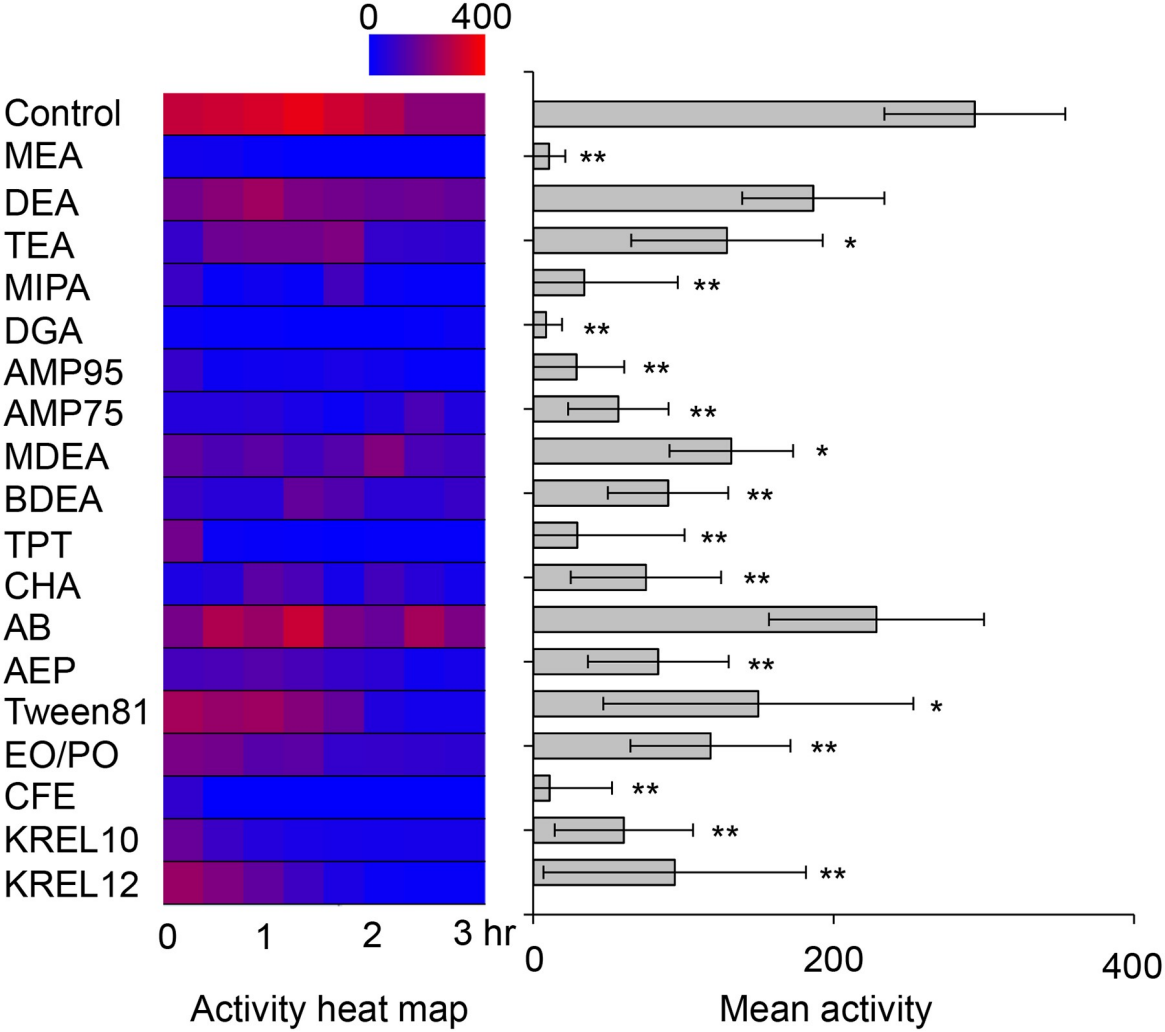
Major constituent amines and surfactants of emulsions exhibited various degrees of toxicity. Zebrafish larvae at 14 dpf were tested at a concentration of 0.5% for amine (v/v) and 0.05% for surfactant. Sample size: *n* = 4 (one animal per well) for amines; *n* = 5 for surfactants. In the heat map, the locomotion activity of all animals tested under the same condition was recorded as a mean value over 5 minutes. * *p* < 0.05; ** *p* < 0.01; *** *p* < 0.001; *p* value, One-way ANOVA with Tukey’s multiple comparison test. The bars and error bars stand for means and standard deviations.

To identify the toxicity factors of emulsions as a mixture, toxicity tests were individually performed on the major constituents of emulsions. A total of 18 ethanolamines and surfactants were chosen and tested using larval fish and a very wide range of toxicities were observed depending on the substance in Fig 3. DEA and AB were the least toxic, whereas MEA, DGA, and CFE were very toxic, and almost no movement was observed after 10 minutes of exposure. The mean activity between DGA and AB was about 28 times greater for the former than the latter. Second, MEA was found to be much more toxic than DEA and TEA. This result indicates that our assay method was sensitive enough to distinguish the degree of toxicity between the simplest ethanolamines. Third, Tween81 was less toxic than other surfactants, which is consistent with the fact that Tween81 is highly biologically stable.

### Chemical structure and concentration affected toxicity

**Fig 4:**
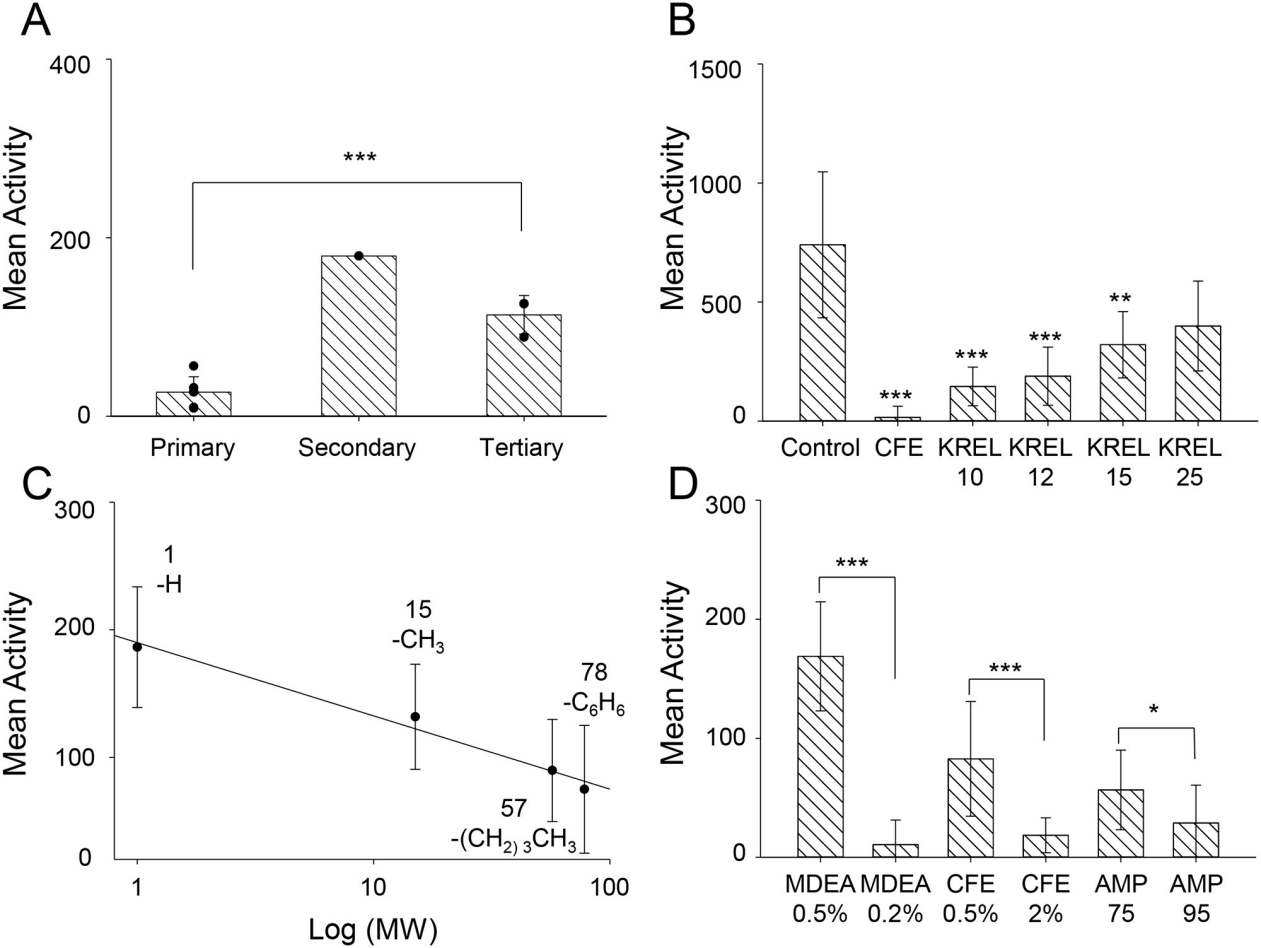
Chemical structure and concentration affect toxicity. (A) Difference in locomotion activity according to the three different types of ethanolamines. Primary (1°) amines are MDEA, MIPA, DGA, and AMP95. The secondary (2°) amine is DEA. Tertiary amines (3°) are TEA, MDEA, and BDEA. (B) Effect of the ethoxylate number of polyoxyethylene castor ether (KREL) on locomotion activity. Sample size: *n* = 10 (one animal per well). The mean locomotion activity of each animal was calculated, and then statistical analysis was conducted. (C) Effect of alkyl chain length of ethanolamines on locomotion activity. ‘-H’, ‘-CH_3_’, ‘-(CH_2_)_3_CH_3_’, and ‘-C_6_H_6_’ indicate DEA, MDEA, BDEA, and CHA, respectively. Zebrafish at 19 dpf were used to identify the more obvious differences in activity value. Sample size: *n* = 10. (D) Effect of dosage concentration on locomotion activity. * *p* < 0.05; ** *p* < 0.01; *** *p* < 0.001; *p* value, One-way ANOVA with Tukey’s multiple comparison test. The bars and error bars stand for means and standard deviations.

Tested ethanolamines can be classified into primary, secondary, or tertiary amines according to the number of carbons attached to nitrogen. MEA, MIPA, DGA, and AMP95 are primary amines whereas TEA, MDEA, and BDEA are tertiary amines. When comparing mean locomotion activity, the primary ethanolamine group was more toxic than the tertiary ethanolamine group (*p* value < 0.001) in Fig 4A. Primary amines are known to be more irritating than secondary or tertiary amines, and our results are consistent with this fact [20]. In another report, the LC_50_ value of MEA in invertebrate was lower than that of MDEA, and the LC_50_ value of MEA was the lowest in algae and bacteria compared to AMP and MDEA [21]. Therefore, comparing the relative toxicity with the LC_50_ value, the MEA is more toxic than the AMP and MDEA. In addition, the LC_50_ values of AMP and MDEA were measured at almost the same level in fish, algae, and bacteria in the report [21], but AMP was found to be more toxic than MDEA in this study. These results may mean that our locomotion analysis method is a very sensitive toxicity analysis method, so we were able to distinguish the relative toxicity between the two chemicals.

The fact that primary amines are more toxic than corresponding secondary or tertiary amines might be attributed to the difference in chemical reactivity. In general, primary amines are more reactive than secondary and tertiary amines [22]. Amines are bioactive substances capable of reacting with the acidic moieties of amino acids, and particularly in primary amines, the lone pair of electrons increases the reactivity with the electrophilic compound [22]. Because of the high reactivity of the primary amines, they appear to be highly toxic since they act directly on proteins and organs and cause more damage.

The ethoxylated number of nonionic surfactants can determine the nature of the chemicals and affect toxicity. We tested CFE and multiple polyoxyethylene castor ethers (KRELs) with different EO moles and identified a clear tendency for toxicity to increase as the EO number decreases in Fig 4B. Animals treated with KREL10 and CFE were also found dead or severely damaged. Thus, our results are consistent with the fact that the lower the number of EO moles, the higher the toxicity [1, 3].

The alkyl chain length or molecular weight (MW) of the secondary di-ethanolamine also had a significant effect on toxicity. DEA and MDEA exhibited less toxicity than BDEA and CHA, indicating that the longer the alkyl chain length or molecular weight, the greater the toxicity in Fig 4C. There was a high correlation between the mean locomotion activity and natural log of molecular weight (R^2^ = 0.9811). This result is consistent with the fact that chemicals with a longer alkyl chain length are more toxic than those with a shorter alkyl chain length [23, 24].

Although we plotted the molecular weight of the branch chemical structure and mean activity to show the correlation between ethanolamine structure and toxicity, the length of the alkyl chain in aminoalkanols affects hydrophobicity. It is known that there is a linear correlation between toxicity and hydrophobicity given as log *K*_*ow*_ (1-octanol/water partition coefficient) [25]. The log *K*_*ow*_ values of DEA and MDEA were -1.43 and -1.04, respectively, and MDEA was higher than DEA. Therefore, there is an inverse correlation between mean activity and log *K*_*ow*_. The lower the mean activity, the higher the toxicity, so this result agrees with the fact that it should be generally proportional to log *K*_*ow*_.

More importantly, the toxicity of the chemical compounds was strongly dependent on their dosage concentration in Fig 4D. MDEA and CFE were much more toxic at 2% concentration than at 0.5%. AMP95 (AMP 95%) was also more toxic than AMP75 (AMP 75%).

### Emulsion particle size might affect toxicity

Although particle size distribution is considered a very important property of emulsions, little is known about the correlation between particle size and toxicity. Thus, its correlation with toxicity was also examined. The D50 values of Oil-A, -B and -C, which were the most toxic, were 313 nm, 1.04 μm and 5.67 nm, and the D50 values of Oil-D, -E and -F, which were the least toxic, were 1.12 μm, 14.5 nm, and 239 nm, respectively. Because of analyzing small numbers of emulsion samples with very different chemical compositions, it was difficult to find the general correlation between toxicity and particle size distribution.

**Fig 5:**
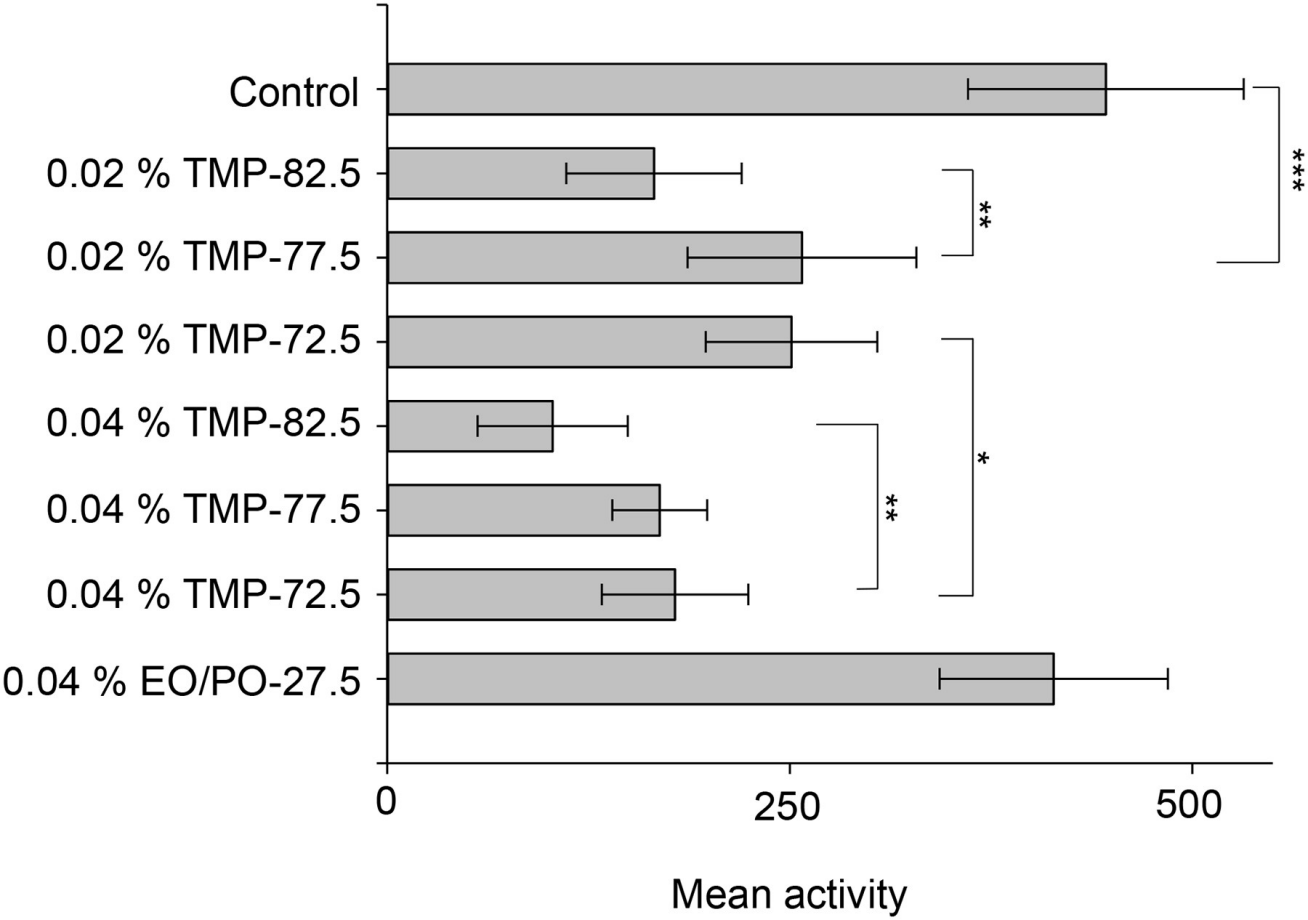
Emulsion particle size affects toxicity. TMP-82.5, which had a bigger emulsion particle size, was more toxic than TMP-72.5 and TMP-77.5. EO/PO-27.5 was a chemical mixture of 72.5% water and 27.5% EO/PO surfactant (w/w). Sample size: *n* = 10 (one animal per well) for TMP-72.5, TMP-77.5, and TMP-82.5; *n* = 5 for EO/PO-27.5 surfactant. * *p* < 0.05; ** *p* < 0.01; *** *p* < 0.001; *p* value, One-way ANOVA with Tukey’s multiple comparison test. The bars and error bars stand for means and standard deviations.

To determine the effect of different emulsion particle sizes on toxicity, emulsion samples with very similar constituents should be prepared. Thus, simple emulsions composed of TMP TO (Trimethylolpropane trioleate) and EO/PO surfactant were prepared and tested for toxicity. TMP-82.5 was more toxic than TMP-72.5 at 0.02% (*p* value = 0.002) and 0.04% (*p* value = 0.021) in Fig 5. TMP-82.5 had a larger emulsion particle size (D50 = 231 nm) than TMP-72.5 (D50 = 168 nm) and TMP-77.5 (D50 = 158 nm). Considering that there was no significant difference in the content of TMP TO in the three TMP emulsions, and EO/PO surfactant was almost non-toxic, the main factor affecting toxicity was thought to be emulsion particle size. Surface tension can also be considered, but there was no significant difference between the TMP emulsions. Further research is needed, but we believe that emulsion particle size can modify the inherited toxicity imparted by the chemical composition although major toxicity is determined by the type and concentration of the constituent chemicals.

### Conclusive statements

An emulsion is a complex two-phase liquid system composed of multiple chemicals, so large-scale experiments at various conditions are required to identify toxic factors. In this study, we developed an imaging-based high-throughput toxicity assay using vertebrate zebrafish. By analyzing the locomotion activity from images, we quantified the relative degree of toxicity of emulsions and their ingredient chemicals with high sensitivity. From the results of large-scale experiments, we not only identified the types and structural properties of chemicals that affect toxicity but also showed size-dependent toxicity of emulsion. Other emulsion properties such as surface tension, emulsion stability, mass transfer, wettability and affinity between emulsion and animal cells may play an important role in toxicity, but little is known about their toxicity. We hope that this study will be the basis for further toxicity studies to help develop reduced-toxicity emulsion products.

## Supporting information

S1 Fig Emulsion particle size distribution. (TIF)

S2 Fig Automated image analysis software. The software was written in VB.NET. After setting the image processing parameters, users can automatically analyze all images through batch processing. The analyzed result can be saved as a text file and draw a heat map.
(TIF)

S3 Fig Sample image analysis results. From binarized images, pixel count, average distance, and improved locomotion activity were calculated.
(TIF)

S4 Fig Comparison between original and new locomotion activities. (A) Correlation between improved locomotion activity and measured travel length. R^2^ = 0.9863. (B) Correlation between pixel count and measured travel length. The sigmoid function with 3 parameters (y = a / (1+e^-(x-xo)/b^) was used for regression. R^2^ = 0.9610.
(TIF)

S1 File Partial programming code to compute the locomotion activity written in VB.NET. (DOCX)
