# Supplementary figures and images for: High-throughput toxicity study of lubricant emulsions and their common ingredients using zebrafish
Source: PLoS One. 2018 Nov 21;13(11):e0207946. doi: 10.1371/journal.pone.0207946 (PMC6249010; doi:10.1371/journal.pone.0207946)

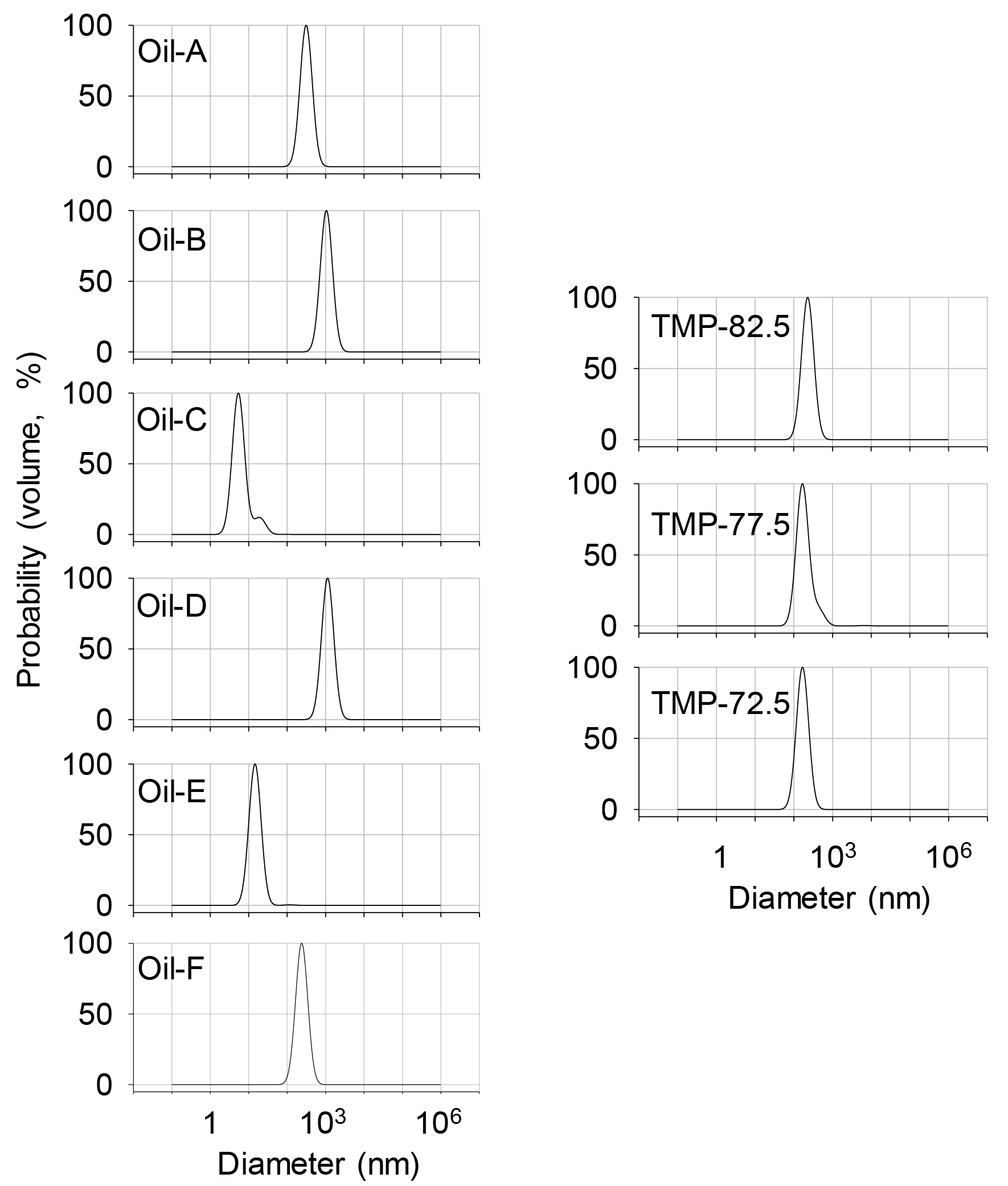

Supplement: S1 Fig — (TIF) [file pone.0207946.s001.tif]

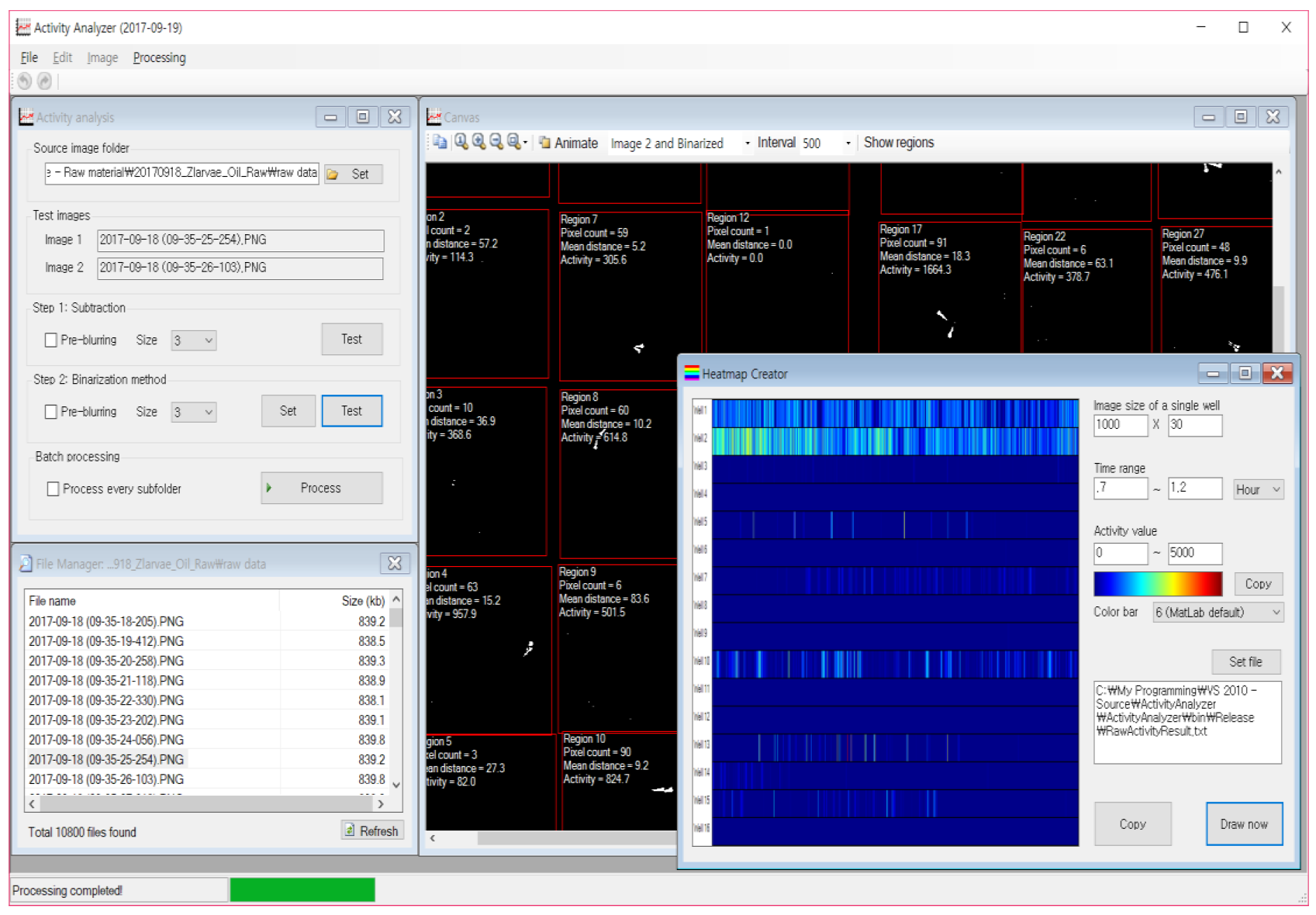

Supplement: S2 Fig — The software was written in VB.NET. After setting the image processing parameters, users can automatically analyze all images through batch processing. The analyzed result can be saved as a text file and draw a heat map. (TIF) [file pone.0207946.s002.tif]

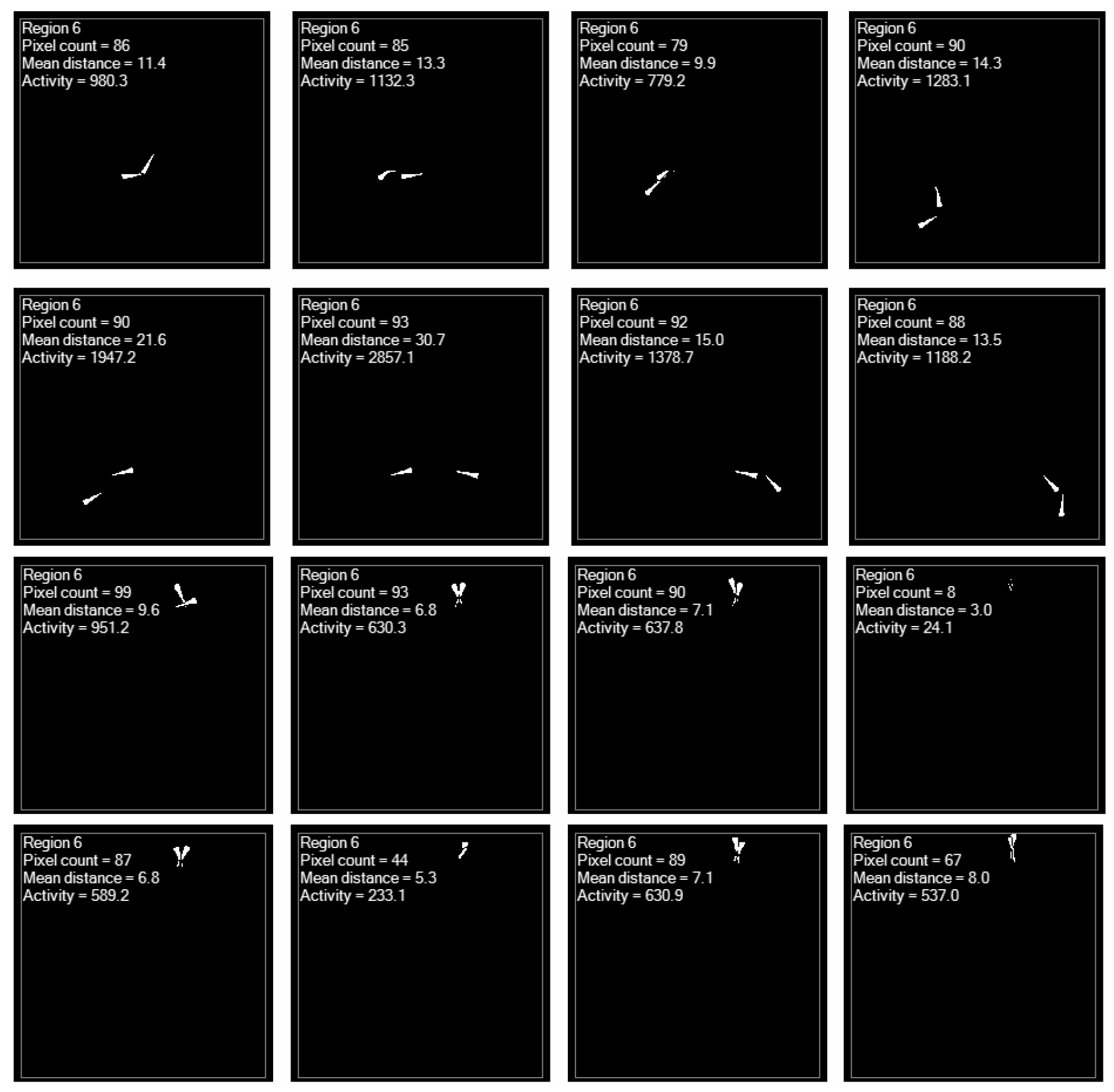

Supplement: S3 Fig — From binarized images, pixel count, average distance, and improved locomotion activity were calculated. (TIF) [file pone.0207946.s003.tif]

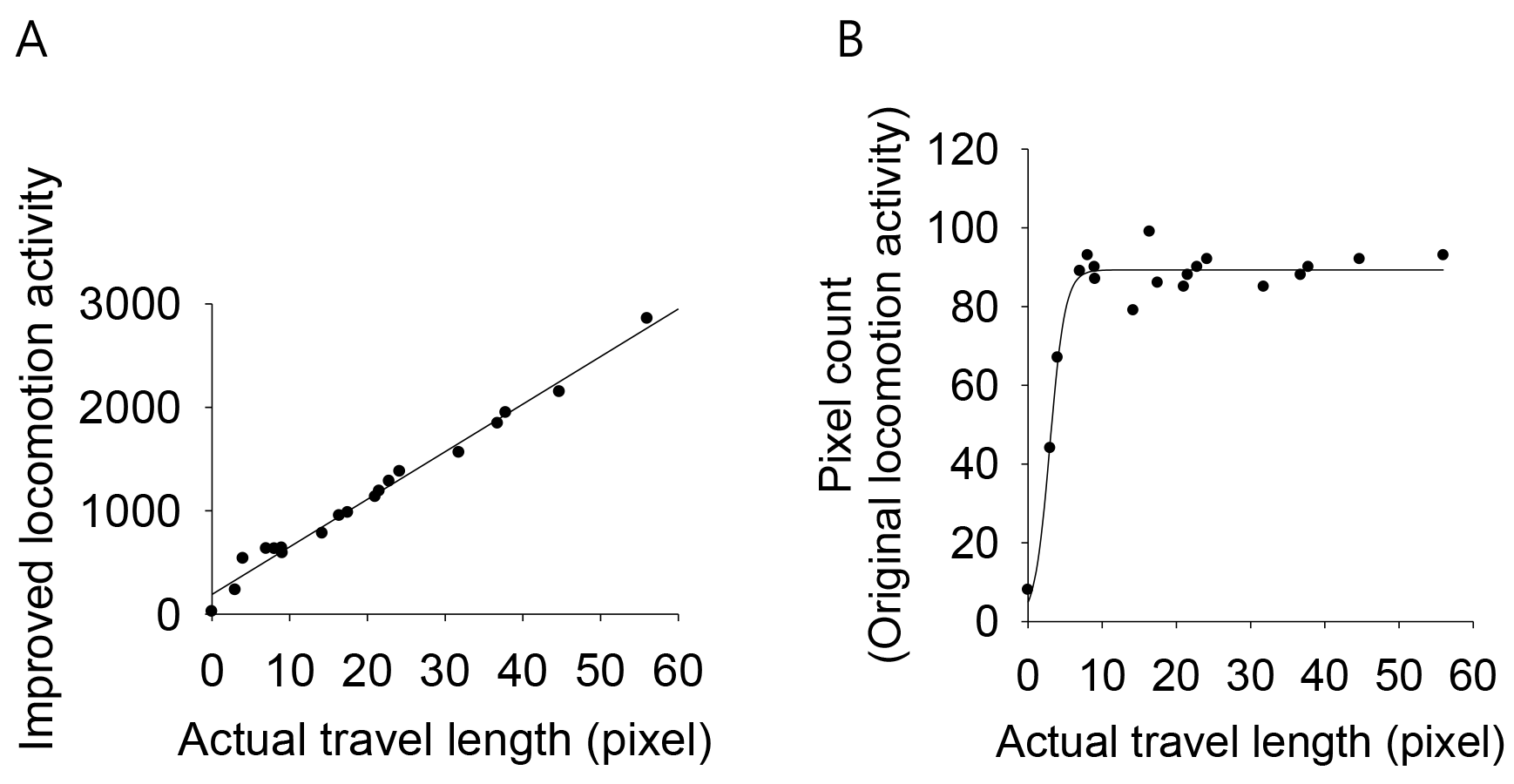

Supplement: S4 Fig — (A) Correlation between improved locomotion activity and measured travel length. R2 = 0.9863. (B) Correlation between pixel count and measured travel length. The sigmoid function with 3 parameters (y = a / (1+e-(x-xo)/b) was used for regression. R2 = 0.9610. (TIF) [file pone.0207946.s004.tif]
