# Supplementary material for: High-throughput toxicity study of lubricant emulsions and their common ingredients using zebrafish
Source: PLoS One. 2018 Nov 21;13(11):e0207946. doi: 10.1371/journal.pone.0207946 (PMC6249010; doi:10.1371/journal.pone.0207946)
Supplement: S1 File — (DOCX) [file pone.0207946.s005.docx]

Partial programming code to compute the locomotion activity written in VB.NET

'Compute locomotion activity of each well

'White_PixelCount indicates the total number of white pixels in the binarized differential image

Public Sub Compute_LocomotionActivity(TargetRectangles() As Rectangle)

'Other parts of the codes were omitted...

AvgDistance(q) = Calculate_AvgDistance(PointArray)

LocomotionActivity (q) = White_PixelCount(q) * AvgDistance(q)

'Other parts of the code are omitted...

End Sub

'XYPoints indicates a point array of white pixels in the binarized differential image

Public Function Calculate_AvgDistance(ByVal XYPoints() As Point) As Double

Dim MeanDistance As Double

Dim SumDistance As Double = 0

Dim DistanceCount As Long = 0

Dim q, r As Integer

Dim XYPointCount As Long

XYPointCount = XYPoints.Count

If XYPoints.Count < 2 Then Return 0

For q = 0 To XYPointCount - 2

For r = q To XYPointCount - 1

SumDistance = SumDistance + Distance(XYPoints(q), XYPoints(r))

DistanceCount = DistanceCount + 1

Next

Next

MeanDistance = SumDistance / DistanceCount

Return MeanDistance

End Function

'Compute distance between two white pixels

Public Function Distance(ByVal XY1 As Point, XY2 As Point) As Double

Dim R As Double

R = Math.Sqrt((XY2.X - XY1.X) ^ 2 + (XY2.Y - XY1.Y) ^ 2)

Return R

End Function
